# Effective prophylaxis against rotavirus diarrhea using a combination of *Lactobacillus rhamnosus *GG and antibodies

**DOI:** 10.1186/1471-2180-7-86

**Published:** 2007-09-27

**Authors:** Neha Pant, Harold Marcotte, Harald Brüssow, Lennart Svensson, Lennart Hammarström

**Affiliations:** 1 Division of Clinical Immunology, Department of Laboratory Medicine, Karolinska Institutet at Karolinska University Hospital Huddinge, SE-141 86 Stockholm, Sweden; 2 Nutrition and Health Department, Food and Health Microbiology, Nestlé Research Centre, CH-1000 Lausanne, Switzerland; 3 Division of Molecular Virology, University of Linköping, SE-581 83, Linköping, Sweden

## Abstract

**Background:**

Rotavirus is a worldwide cause of infectious infantile diarrhea that claims over 600,000 lives annually. Recently, two new vaccine candidates have been developed but their efficacy in developing countries, still remains to be proven. Oral delivery of specific immunoglobulins provides passive immunity and is a fast acting treatment for rotavirus diarrhea. Probiotic bacteria have also gained considerable attention lately as treatment for rotavirus diarrhea. Here we report an evaluation of the therapeutic potential of different probiotics and their combination with anti – rotavirus antibodies in a mouse model of rotavirus diarrhea.

**Results:**

Of the six probiotic bacteria tested, *Lactobacillus rhamnosus *strain GG had the strongest influence in reducing prevalence, duration and severity of diarrhea and was therefore chosen for combination treatment with immunoglobulins. The combination treatment reduced the diarrhea outcome measures significantly, prevented histopathological changes and reduced the virus load in the intestines.

**Conclusion:**

The advantages associated with immunoglobulins and probiotics based therapy is that the treatment provides a rapid therapeutic effect and is cost efficient. These components do not require special storage conditions and could potentially complement the rehydration therapy that is currently used.

## Background

Diarrheal diseases remains a major global threat to child survival [1], and rotavirus is the predominant agent of severe, dehydrating gastroenteritis in infants and young children in both developing and industrialized countries [2,3]. In the Western world, it accounts for a major economical loss with a yearly cost of over $ 1 billion in the management of rotavirus infection in the US alone [4]. The recent development of two new rotavirus vaccines offers hope but even if an effective vaccine becomes available, its use may be limited by financial constraints in developing countries. Moreover, its efficacy in children with malnutrition and associated immunodeficiency is questionable. In the absence of an ideal and affordable specific treatment, Oral Rehydration Therapy (ORT) has served as a useful treatment that may be rapidly distributed, does not require specific storage conditions and is inexpensive. However, even after achieving a substantial reduction in mortality from dehydration, ORT has little or no effect on the course of diarrhea or its associated nutritional morbidity.

The role of secretory IgA, in serving as the first line of defense against many mucosal pathogens is well established. In the case of rotavirus, protection from disease appears to rely mainly on the production of neutralizing antibodies against the outer capsid proteins VP4 and VP7[5]. As a corollary to this, oral delivery of specific antibodies protects against severe rotavirus diarrhea both in laboratory and clinical settings [6]. We have previously demonstrated that purified antibodies from hyperimmune bovine colostrum and egg yolk from appropriately immunized hens are effective in the treatment of diarrhea in rotavirus-infected children [7,8]. However, mass prophylaxis with HBC has logistic and economic limitations, particularly in developing countries.

In the last few decades, the use of probiotic bacteria has gained considerable attention as a safe and accessible form of treatment for gastrointestinal diseases [9,10]. Bacteria that have been employed for intervention of diarrhea of viral or bacterial origin belong to the *Lactobacillus *or the *Bifidobacterium *genus [11]. The therapeutic capacity of certain probiotic bacteria against rotavirus gastroenteritis has been suggested to be due to their ability to stabilize and reinforce the mucosal barrier [12], production of antimicrobial substances [13] and stimulation of the local antigen specific and nonspecific immune responses [14,12]. Significant differences have also been noted with regard to the efficaciousness and mode of action of different strains.

The purpose of our study was to evaluate a combination therapy with immunoglobulins and probiotics as a prophylaxis against rotavirus infection in a mouse model.

## Results

### Reactivity of HBC preparation with RRV

**Figure 1:**
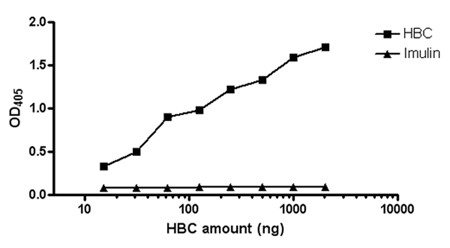
**Reactivity of Hyperimmune bovine colostrums (HBC) against RRV**. HBC preparation is highly reactive against RRV as assessed by ELISA. ELISA plates were coated with RRV and HBC was added in different dilutions. The reaction was developed using anti-bovine AP conjugated secondary antibody. Control colostrums preparation (Imulin^®^) does not show any cross-reactivity with RRV even at high concentrations.

HBC (Hyperimmune Bovine Colostrum) antibodies were highly reactive against RRV (Rhesus rotavirus) in ELISA, even at low concentrations (15 ng of total protein, corresponding to 5.4 ng of total immunoglobulins). A control colostrum preparation Imulin^®^, did not show any reactivity against RRV (Figure 1).

### *In-vitro *neutralization test

Since the preparation of the anti-rotavirus HBC used has had a shelf life of nearly 20 years, it was important to evaluate its neutralization capacity against RRV, our challenge pathogen. MA104 cells grown to confluency were thus challenged with a fixed amount of RRV (200 FFU) after the virus had been preincubated anti-rotavirus HBC. Even a low amount (7 ng total protein of anti-rotavirus HBC corresponding to 2.5 ng total immunoglobulins) afforded 100% protection of the challenged cells (data not shown).

### Evaluation of immunoglobulin and probiotic combinations on rotavirus diarrhea

The anti-rotavirus HBC preparation was highly effective in preventing diarrhea in pups challenged with rotavirus. Daily administration of 100 μg/dose of anti-rotavirus HBC (36 μg/dose of immunoglobulins) resulted in a 75% decrease in diarrhea prevalence on day 2 and 84% on day 3 compared to the percentage prevalence in infected but untreated mice (p = 0.0047 for day 2 and 0.0007 for day 3). Diarrhea duration and severity was also reduced significantly from a score of 1.85 and 3.0 in infected and untreated group to 0.42 for both parameters in mice receiving 100 μg anti-rotavirus HBC (p < 0.001 for both). A dose of 10 μg HBC (Table 2) or lower (1 μg or 100 ng HBC) (data not shown), did not impart any protection against rotavirus challenge.

**Table 2:** Duration and severity of diarrhea in different treatment groups with or without complementation with anti-rotavirus HBC

Groups	Number of animals	Duration (mean ± SE)*	Severity (mean ± SE)
Untreated	27	1.85 ± 0.17	3.00 ± 0.33
100 μg HBC	12	0.42 ± 0.23***	0.42 ± 0.23 ***
10 μg HBC	8	1.62 ± 0.32	2.62 ± 0.53
*L. paracasei*^b^	10	1.60 ± 0.27	^a^
*L. paracasei*^b ^+ 10 μg HBC	9	0.78 ± 0.28 *	^a^
*L. reuteri SD 2112*	10	1.20 ± 0.20	2.00 ± 0.47
*L. reuteri SD 2112 *+ 10 μg HBC	10	1.20 ± 0.20	2.00 ± 0.47
*L. paracasei *NCC 2461	10	1.80 ± 0.29	2.50 ± 0.40
*L. paracasei *NCC 2461+ 10 μg HBC	7	2.14 ± 0.26	3.43 ± 0.53
*L. rhamnosus GG*	10	0.90 ± 0.23	1.20 ± 0.32*
*L. rhamnosus GG *+ 10 μg HBC	8	0.62 ± 0.32*	0.87 ± 0.48 *

*** p < 0.001, * p < 0.05 Vs. Untreated

^a ^absolute values for severity are not available, but they are at least as much as the duration.

^b ^previously thought to be *L. casei *ATCC 393 [15]

**Figure 2:**
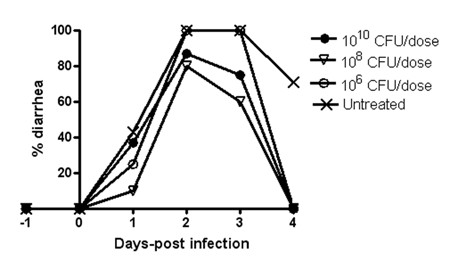
**Optimization of the dose for oral treatment with probiotic bacteria using *L. paracasei *[15] as a reference strain**. *L. paracasei *[15] was fed in different doses and the pups challenged with RRV on day 0. 10^8 ^CFU of bacteria was selected as the optimal dose for subsequent treatment with probiotic bacteria.

For subsequent development of combination treatments, we started by optimizing the dose of probiotic bacteria being fed, using *Lactobacillus paracasei *as a reference [15]. The strain was previously considered a *Lactobacillus casei *ATCC 393^T ^variant cured of plasmid pLZ15 and has been referred in the past as *L. casei *or *Lactobacillus zeae *ATCC 393 (pLZ15^-^). Recently, using molecular techniques, it has correctly been identified as *L. paracasei*. For simplicity, we will refer to this strain as *L. paracasei *[15] in this article. This strain is 'molecularly accessible' and has previously been used for heterologous protein expression [16]. Mouse pups were fed three different doses of *L. paracasei *[15] (10^10^, 10^8 ^or 10^6 ^CFU/dose) and challenged with RRV. The daily administration of the bacteria at 10^8 ^CFU resulted in a small, statistically non-significant reduction in diarrhea prevalence. However, the dose of 10^10 ^CFU was not significantly better than 10^8 ^CFU and we thus decided on using the lower dose, 10^8 ^CFU as the standard dose for subsequent experiments (Figure 2).

In total, bacterial strains from 6 different *Lactobacillus *species were evaluated for their prophylactic action against RRV-induced diarrhea in our mouse model. The cumulative results of the outcome measures are summarized in Table 1. *L. paracasei *[15] induced a modest (non-significant) reduction in diarrhea prevalence to 65% and no significant improvement in diarrhea duration or severity was observed. *Lactobacillus rhamnosus *strain GG showed a strong anti-rotavirus capacity and reduced the diarrhea prevalence to 41% compared to 93% in infected but untreated mice (p < 0.001) (Table 1). The diarrhea duration and severity was also significantly reduced (p <0.001). The 'anti-rotavirus' activity of the bacteria is dependant on viability or is destroyed during heat inactivation, as heat inactivated *L. rhamnosus *GG did not impart protection in mice (Table 1). *Lactobacillus reuteri *strain SD2112 conferred a small but non-significant reduction in diarrhea prevalence, duration and severity (Table 1).

**Table 1:** Outcome measures of diarrhea among mice receiving different probiotic bacteria and challenged with RRV

**Strains/Treatment ^a^**	**Number of animals**	**Highest Prevalence (%)**	**Duration (mean ± SE)**	**Severity (mean ± SE) **
*L. paracasei *^b^	17	65	1.94 ± 0.20	2.88 ± 0.35
*L. reuteri *SD2112 (ATCC 55730)	10	70	1.40 ± 0.30	2.00 ± 0.47
*L. rhamnosus *GG (ATCC 53103)	20	41***	0.65 ± 0.15***	0.85 ± 0.19***
Heat killed *L. rhamnosus *GG (ATCC 53103)	8	75	1.63 ± 0.32	2.37 ± 0.53
*L. paracasei *NCC 2461 (ST11)	10	90	1.60 ± 0.26	2.50 ± 0.40
*L. johnsonii *NCC 533 (La-1)	8	88	1.87 ± 0.12	2.75 ± 0.25
*S. thermophilus *NCC 2496	10	100	1.50 ± 0.16	2.70 ± 0.39
Infected and untreated	27	93	1.81 ± 0.17	2.96 ± 0.32

^a ^All bacteria were daily administered at a concentration of 10^8 ^CFU/dose

^b ^previously thought to be *L. casei *ATCC 393 [15]

* p < 0.05 vs Untreated

**p < 0.01 vs Untreated

*** p < 0.001 vs Untreated

**Figure 3:**
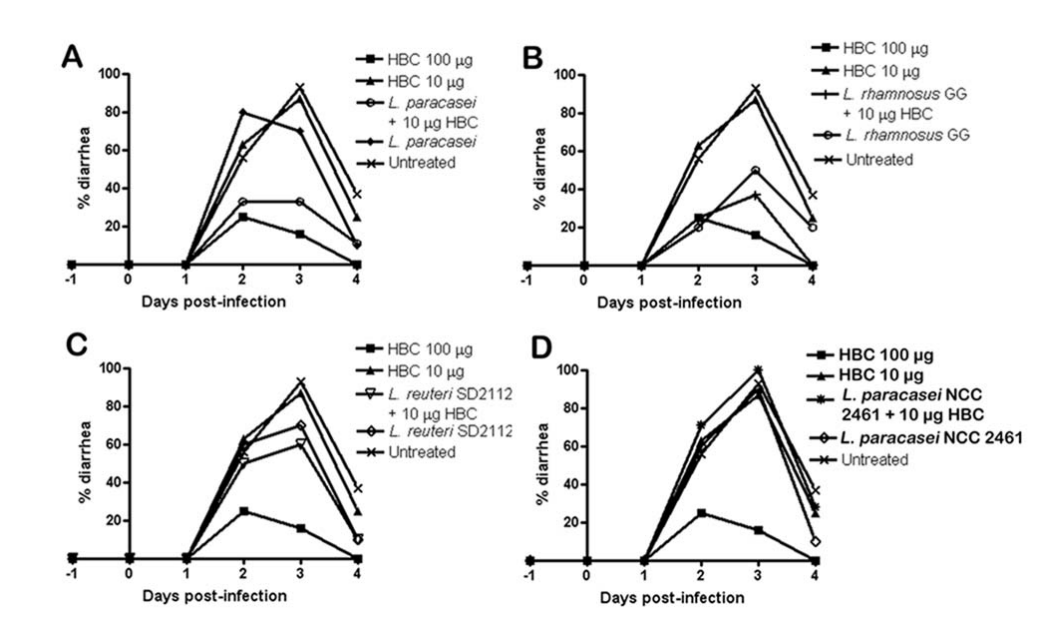
**Anti-rotavirus HBC combination treatment with different species of *Lactobacilli***. Mice were fed lactobacilli daily, either alone or supplemented with 10 μg anti-rotavirus HBC and challenged with RRV on day 0. Diarrhea prevalence was recorded every day and is presented as percentage prevalence. Test of significance was performed using Fischer's exact test. **(A) ***L. paracasei *[15], in combination with HBC, was able to achieve a 58% better protection than that imparted by bacteria alone on day 2. On day 3 the combination treatment achieved statistically significant reduction in diarrhea prevalence in comparison to infected but untreated mice (p = 0.001) and 53% better protection than *L. paracasei *[15] alone. **(B) ***L. rhamnosus *GG alone caused a significant reduction in diarrhea prevalence in comparison to untreated mice on day 3 (p = 0.009) and in combination with HBC this effect was further enhanced (p = 0.003). **(C) **On day 3, protection conferred by combined therapy of *L. reuteri *strain SD2112 with 10 HBC μg was statistically significant in comparison to untreated mice (p = 0.035). **(D) **Administration of either *L. paracasei *strain NCC 2461 alone or supplemented with anti-rotavirus HBC did not change the diarrhea profile which resembled that of the untreated mice.

It was previously noted that anti-rotavirus HBC at 10 μg/dose could not protect against development of severe rotavirus diarrhea. We therefore used HBC at this dose to screen for additive or synergistic combinations with probiotic bacteria. *L. paracasei *[15], *L. reuteri *SD2112 and *Lactobacillus paracasei *strain NCC 2461 (referred to as ST11 in (23)) were selected along with *L. rhamnosus *GG to develop the combination treatment. Complementation of *L. paracasei *[15] or *L. rhamnosus *GG with HBC was more protective than either bacteria given alone. Combination of *L. paracasei *[15] and HBC reduced the diarrhea prevalence to 33% on day 2 and 3 whereas it remained high (70–80%) in *L. paracasei *[15] only treated mice (relative reduction of 58% on day 2 and 53% on day 3). The diarrhea duration was also significantly reduced in the combination group (p <0.05) (Figure 3A). As observed previously, *L. rhamnosus *GG could itself induce a statistically significant reduction in diarrhea prevalence in challenged mice on day 3 (p = 0.009) and mollified disease severity (p < 0.05). Nonetheless, the combination of bacteria with 10 μg anti-rotavirus HBC caused a further 26% relative reduction in diarrhea prevalence on day 3 and significantly reduced the duration and severity of the disease (p < 0.05) (Figure 3B). Combination of *L. reuteri *SD 2112 and HBC did not show the same efficacy in reducing diarrhea but achieved a modest improvement over *L. reuteri *SD2112 given alone on both day 2 and 3 (16% and 14% relative reduction, p = 0.035 for day 3) (Figure 3C). *L. paracasei *NCC 2461 by itself, or in combination with HBC, did not protect against RRV-induced diarrhea (Figure 3D and Table 2).

### Histopathological analysis

**Figure 4:**
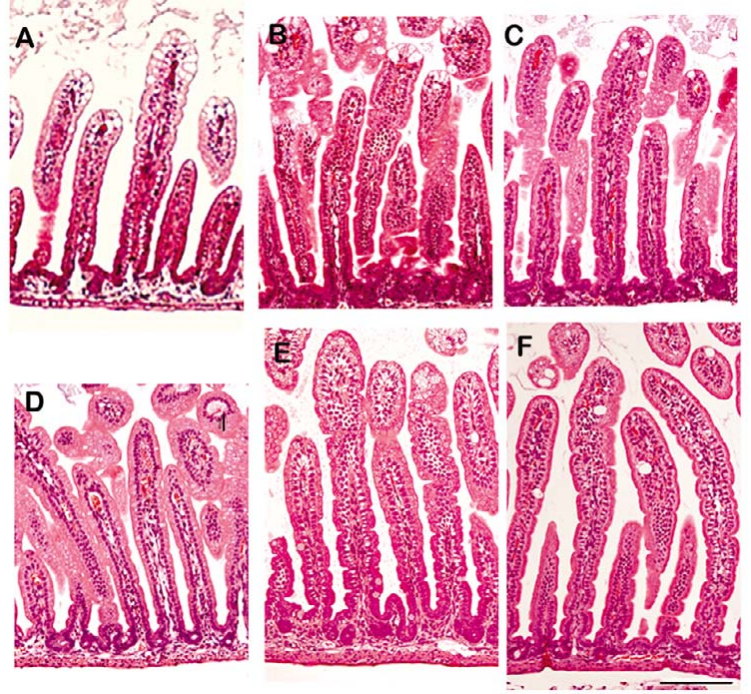
**Hematoxylin/Eosin stained sections of jejunum from mice treated with different formulations**. Tissue sections were excised and embedded in paraffin. HE staining was performed by standard protocols and the samples assessed blindly for signs of rotavirus infection. **(A) **infected and untreated mice shows typical signs of grave rotavirus infection with swollen and vacuolized villus tips. **(B) ***L. rhamnosus *GG treated mice show moderately resolved histopathology similar to **(C) **10 μg/dose HBC treated mice. **(D) ***L. rhamnosus *GG combined with HBC is able to resolve the histopathology to normalcy. **(E) **no histopathology associated with treatment with 100 μg/dose HBC. (F) uninfected control mice.

Formalin fixed intestinal tissue sections from mice treated with different treatment modalities were blindly analyzed for histopathological changes associated with rotavirus infection [17]. The RRV infected, untreated group presented a typical histology associated with rotavirus infection with swollen villus tips and vacuolization. The villus tips were unstainable due to epithelial cell death (Figure 4A). The histo-pathology showed reduced vacuolization in pups receiving *L. rhamnosus *GG or 10 μg HBC alone (Figure 4B and 4C). In comparison, the combined treatment of *L. rhamnosus *GG with 10 μg of HBC prevented histological changes completely (Figure 4D) as was also seen with 100 μg of HBC (Figure 4E). The negative control mice that were not infected showed a normal histology (Figure 4F).

### Real Time PCR

**Figure 5:**
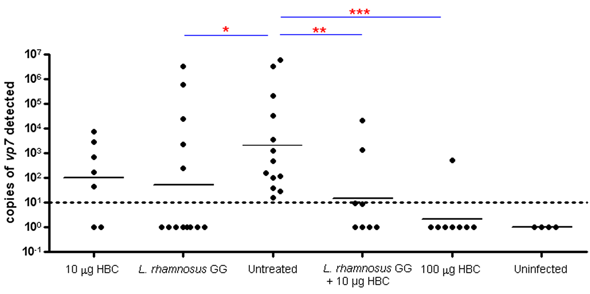
**Real time PCR of intestinal tissue sections for RRV *vp7 *gene**. Tissue samples were excised from small intestines and total cellular RNA was extracted. Real time PCR was performed for rotavirus *vp7 *gene. The bars represent geometric mean of the virus load after normalization with housekeeping *gapdh *gene. The combination of 10 μg HBC and *L. rhamnosus *GG was able to achieve a statistically significant reduction of virus load (as tested by Fischer's test). *** p < 0.0001, ** p = 0.0005, * p = 0.0016

Since *L. rhamnosus *strain GG based therapy (either only bacteria or combined with HBC) significantly reduced diarrhea prevalence among the challenged mice and also reduced the associated histopathological changes, we wanted to confirm whether this therapy also had an effect on the virus load. Total cellular RNA isolated from intestinal tissue sections of pups treated with *L. rhamnosus *strain GG based treatment modalities (bacteria alone or combined with 10 μg HBC) were therefore analyzed by real time PCR for number of copies of the RRV *vp7 *gene. The virus load in infected, untreated mice was higher than in all the treatment groups (geometric mean 2038). As expected, no virus could be detected in uninfected negative control mice. Treatment with 100 μg of HBC was able to reduce the virus load in challenged mice well below the detection level of 10 *vp7 *copies and hence, corroborated the normal histology (geometric mean 2) (p < 0.0001). In comparison, the lower dose of 10 μg could not achieve a similar clearance of virus and the load was still high with a geometric mean of 102. Treatment with *L. rhamnosus *GG alone was also able to reduce the virus load significantly (geometric mean 53) (p = 0.001). The combined treatment of 10 μg of HBC and *L. rhamnosus *GG resulted in a statistically significant reduction of virus load in comparison to infected and untreated mice (geometric mean 15) (p = 0.0005) (Figure 5).

## Discussion

One of the advantages of using immunoglobulin-based treatments against rotavirus-induced diarrhea is the immediacy of action, which the conventional active immunization regimens lack. We have previously reported that severe rotavirus diarrhea in children can be successfully treated using antibodies derived from colostrum of immunized cows (HBC antibodies) [7,18,19]. Our preliminary results from this study establishes that these antibodies have been well preserved even after a long shelf life (18 years) and are fully functional in imparting protection against rotavirus infection *in vitro *and *in vivo*. However, treatment for rotavirus diarrhea purely based on HBC, however effective, is not practical and may be offset by the costs involved for mass prophylaxis. It is thus imperative to find alternative methods to make immunoglobulin based therapy economically viable.

Probiotic bacteria offer a cheaper platform for the management of rotavirus diarrhea. Among the various mechanisms suggested for the action of probiotics is the ability to survive gut transit and in the process cause stabilization of the mucosal barrier, production of anti-microbial compounds and stimulation of the mucosal immune response leading to an increase in secretory IgA [14]. It is, however, difficult to determine whether the health promoting effects of the probiotic bacteria are due to a specific inhibition of the pathogen in question or a reflection of a more complex interaction between host, pathogen and the probiotic.

We tested six different lactobacilli for protection against rotavirus-induced diarrhea. Mouse pups received two prophylactic doses of bacteria before challenge with rotavirus, followed by daily therapeutic administration of respective bacteria and monitoring for diarrhea symptoms. We noted a strong anti-rotavirus activity of *L. rhamnosus *strain GG, corroborating results obtained previously by other researchers in clinical trials [20,21]. However, the same bacterium, when heat killed, was unable to impart protection against rotavirus challenge, suggesting that the inhibitory effect against rotavirus is either dependent on viability or is a heat labile factor. Interestingly a clinical evaluation of treatment with live or heat inactivated *L. rhamnosus *GG against rotavirus diarrhea found that the inactivated bacteria did not effectively stimulate local IgA production, thus increasing the chances of reinfection [14]. *L. reuteri *SD2112, which has previously shown a positive effect in the treatment of rotavirus-related diarrhea in clinical trials [22], was only marginally protective in our mouse model and *L. paracasei *NCC 2461, which was recently shown to have no significant effect in resolving rotavirus-induced gastroenteritis [23], was not protective at all in our animal model. Thus, it appears that the basic mechanism of probiotics, at least in relation to rotavirus infection, may be paralleled in the mouse gastro-intestinal tract as in the humans and the mouse model may thus potentially serve as a premonitory assessment of the therapeutic effect of candidate probiotics against rotavirus.

In order to introduce specificity to the basic probiotic therapy, we complemented bacteria with a low dose of polyclonal anti – rotavirus antibodies. Four lactobacilli with wide ranging anti-rotavirus properties were administered in combination with antibodies. Combination treatment of *L. rhamnosus *GG and antibodies was the most potent among all the combinations. Not only did this combination reduce the outcome measures of diarrhea, which was also partially achieved by feeding of *L. rhamnosus *GG alone, but also alleviated histopathology and reduced the virus load in the small intestine. The role of secretory IgA and passively administered antibodies as the primary protection against many invading mucosal pathogen including rotavirus is an established tenet. In fact, the probiotic activity of *L. rhamnosus *GG with respect to rotavirus infection has been linked to an increased rate of production of IgA in the mucosa [14]. We also attempted to study the possible contribution of *L. rhamnosus *GG in stimulating IgA responses in the gastrointestinal mucosa of the pups through ELISA. However, such an analysis is technically difficult, due to the high background by maternally derived IgA through milk. We hypothesize that by administering antibodies and *L. rhamnosus *GG together, we have achieved a combination where the infection is effectively controlled by the two components. The small amount of administered antibodies blunts the initial infection, but is clearly not enough to abrogate it. The co-administered *L. rhamnosus *GG may potentially boost this protection owing to its ability to muster a local IgA response.

To this end, it is reasonable to assume that the cornerstone in achieving protection against rotavirus infection could be to optimize delivery of functional antibodies to the intestinal mucosa. Genetically engineering lactobacilli for *in-situ *expression of antibody fragments could achieve this objective [16,24]. Multiple antibody specificities can be expressed by the lactobacilli that can mimic a polyvalent antibody preparation. The expression of various antibody fragments on lactobacillus surface would increase the avidity for binding several fold. Additionally, the probiotic activity associated with the carrier lactobacilli would be an added advantage. We are thus currently developing lactobacilli that express anti-rotavirus antibody fragments on the bacterial surface.

Problems such as high production costs, special storage conditions, difficulties in distribution to the affected population and the need of technical expertise for vaccine delivery constitute potential drawbacks of active immunization. Furthermore, efficacy of the newly licensed vaccines in the affected population and the recommended use only in children younger than 3 months are issues that need to be addressed. As a principle, inducing a protective response by vaccination would take longer than the time between exposure to rotavirus and the onset of disease. Passive immunization with protective antibodies is the only currently available intervention that provides immediate protection in persons with impaired immunity. Freeze-dried immunoglobulins and probiotics could also be used as a prophylactic measure when a seasonal dependent rotavirus outbreak in suspected. Although the current treatment was administered as a prophylaxis in this study, both *L. rhamnosus *GG and oral immunoglobulin therapy have been previously shown to have therapeutic effect against rotavirus diarrhea in children [20,21,7]. A combination based on these two components may therefore also have therapeutic efficacy but this hypothesis needs further testing. Freeze-dried immunoglobulins and probiotics could be used to complement the standard oral rehydration therapy and may help to relieve the immense disease burden posed by rotavirus in the developing world.

## Conclusion

A combination of *L. rhamnosus *GG with specific bovine colostrum – derived immunoglobulins is an effective prophylactic measure for rotavirus diarrhea in the infant mouse model. This can be a highly cost efficient way of managing rotavirus diarrhea and may thus represent the prophylaxis of choice for selected group of children.

## Methods

### Anti – rotavirus antibodies

The Hyperimmune Bovine Colostrum (HBC) used was produced by vaccination of pregnant cows in a Swiss dairy farm with human strains of rotavirus, i.e. Wa, RV3, RV5 and ST3, representing serotypes 1 to 4. The preparation and the antiviral activity of the HBC concentrate is described in detail elsewhere [19]. In brief, the concentrate was prepared from colostrums by skimming, pasteurization and removal of milk fat, casein, lactose and mineral salts. The product was then sterile filtered, and the resulting whey protein solution was freeze-dried. The immunoglobulin concentration of the powder was 36 g/100 g of dried anti-rotavirus milk concentrate. The compositions of the immunoglobulin were 75% IgG1, 3% IgG2, 17% IgA and 6% IgM. The neutralization titers as measured in type neutralization test against the serotype were as follows: serotype 1, 7500; serotype 2, 2000; serotype 3, 4500; and serotype 4, 4500 [19]. The negative control was prepared from milk from nonimmunized cows (Imulin^®^; New Zealand Dairy Board, Wellington, New Zealand) (22% lactose). Both the HBC and placebo were stored at room temperature.

### Virus cultivation

Rhesus rotavirus (RRV) was cultured in MA104 cells as previously described. The virus titre was calculated using immunoperoxidase staining of infected cells [25]. A single batch of the RRV was used for the study.

### Reactivity of HBC against RRV (ELISA)

ELISA 96 well plates were coated with rabbit anti-human rotavirus anti-serum (1:1000) at 4°C overnight. After two washes with PBS, diluted RRV was added (1:100) and left for binding overnight at 4°C. The plates were then blocked with PBS-BSA (2%) for 2 h and subsequently, HBC was added in two-fold dilutions prepared in PBS and the plate was incubated at 37°C for 1 h. After washing, alkaline phosphatase conjugated anti-bovine IgG was added (1:1000) (Zymed, San Francisco, USA) and the plate was incubated for another 1 h at 37°C. The reaction was developed with p-nitrophenyl phosphate in diaethanolamine buffer and the plate was read at 405 nm.

### Neutralization assay

HBC was serially diluted four-fold in Dulbecco's PBS and incubated for 1 h at room temperature with 200 foci forming units (FFU) of trypsin-activated RRV in a final volume of 200 μl. Confluent MA104 cell monolayers were then inoculated with the mixture. The inoculated plates were incubated at room temperature for 1 h, washed with MEM medium, supplied with fresh MEM medium supplemented with antibiotics (gentamycin, penicillin and streptomycin) and incubated at 37°C in a 5% CO_2 _atmosphere for 18 h. Monolayers were fixed and stained with immunoperoxidase as previously described [25]. A reduction in the number of RRV-infected cells greater than 60% with respect to the number in control wells was considered to indicate neutralization [5].

### Bacterial strains and growth conditions

Four different Lactic acid bacteria were obtained from Nestec, Nestlé, Lausanne, *L. paracasei *strain NCC 2461 (ST11), *L. rhamnosus *strain GG (ATCC 53103), *L. johnsonii *strain NCC 533 (La-1) [26]*, L. rhamnosus *strain NCC 596 and *Streptococcus thermophilus *strain NCC 2496. The *L. reuteri *strain ATCC 55730 (SD2112) was obtained from Biogaia, Sweden. *L. paracasei *[15] (previously named *L. casei *393 pLZ15-) [15] was obtained from Peter Pouwels (TNO Institute, the Netherlands). Lactobacilli were reconstituted and cultured in MRS broth (Difco, Sparks, MD, USA) in standing aerobiosis conditions at 37°C. *S. thermophilus *strain NCC 2496 was cultured in M17 media with lactose supplementation in standing aerobiosis at 42°C.

### Animal Experiments

All animal experiments were approved by the local ethical committee of the Karolinska Institutet at Karolinska University Hospital in Huddinge. Pregnant BALB/c mice were purchased from Møllegard, Denmark. Four-day-old pups were used for the study. Different bacteria or antibody preparations were administered to pups once daily in a 10 μl volume starting on day -1 and continued until day 3. Infections were made orally on day 0 using 2 × 10^7 ^FFU RRV in a 10 μl volume. Immunoglobulins and freshly cultured bacteria were resuspended in PBS at the desired concentrations. Occurrence of diarrhea was recorded daily until day 4. Pups were euthanized using intra-peritoneal pentobarbital on day 4. Sections of small intestines were stabilized in RNAlater^® ^(Qiagen, Hilden, Germany) for RNA isolation or fixed in neutral buffered formalin for histopathological analysis.

### Histopathological Analysis

Sections of small intestine were excised and perfused with formalin. The sections were kept immersed in formalin for a day after which they were transferred to 70% ethanol. The samples were embedded in paraffin and sections were stained with hematoxylin and eosin using standard protocols. The sections were analyzed blindly for signs of rotavirus infection associated pathology [17].

### Real time PCR

Total cellular RNA was isolated from small intestinal tissue and used for real-time PCR analysis after digestion of residual genomic DNA using RNase free DNase^® ^(Qiagen, Hilden, Germany). EZ RT-PCR^® ^core reagent kit (PE Applied Biosystems) was used for real- time PCR quantification of rotavirus *vp7 *RNA as described before [16]. The RNA samples from each animal were normalized for the internal housekeeping gene GAPDH [27]. Detection of no virus or less than 10 virus *vp7 *RNA copies by PCR was defined as clearance from infection.

## Statistics

Diarrhea in the pups was assessed on the basis of consistency of feces. Watery diarrhea was given a score of 2 and loose stool was given a score of 1, no stool or normal stool was given a score of 0. Presence or absence of diarrhea was compared among treatment groups in a day-wise manner by Fischer's exact test and was presented as percentage diarrhea in graphs. Severity was defined as the sum of diarrhea scores for each pup during the course of the experiment (severity = Σ diarrhea score (day 1 + day 2 + day 3 + day 4)) and duration was defined as the total sum of days with diarrhea. Both severity and duration were analyzed by Kruskal-Wallis and Dunn tests. Differences in the intestinal virus load as assessed by real-time PCR were tested using the Mann-Whitney test.

## Competing interests

The author(s) declares that there are no competing interests.

## Authors' contributions

NP carried out the neutralization assay, animal experiments and real time PCR for rotavirus *vp7 *gene. HM carried out statistics and participated in the analysis of data. LS generated RRV for the study and assisted with the neutralization assay. HB and LH conceived of the study and participated in its design and coordination. All authors read and approved of the final manuscript.
